# Meniscal Bearing Dislocation of Unicompartmental Knee Arthroplasty with Faint Symptom

**DOI:** 10.1155/2015/217842

**Published:** 2015-06-07

**Authors:** Tadashi Fujii, Yoshio Matsui, Marehoshi Noboru, Yusuke Inagaki, Yoshinori Kadoya, Yasuhito Tanaka

**Affiliations:** ^1^Department of Orthopaedic Surgery, Kashiba Asahigaoka Hospital, Kaminaka 839, Kashiba, Nara 639-0265, Japan; ^2^Department of Orthopaedic Surgery, Osaka Rosai Hospital, Nagasone-cho 1179-3, Kita-ward, Sakai, Osaka 591-8025, Japan; ^3^Department of Orthopaedic Surgery, Nara Medical University, Shijo-cho 840, Kashihara, Nara 634-0813, Japan; ^4^Department of Orthopaedic Surgery, Hanwa Daini Senboku Hospital, Toyoda 1588-1, Minami-ward, Sakai, Osaka 590-0106, Japan

## Abstract

We experienced two cases of atypical lateral dislocations of meniscal bearing in UKA (unicompartmental knee arthroplasty) without manifest symptoms. The dislocated bearing, which jumped onto the wall of tibial components, was found on radiographs in periodic medical examination although they could walk. Two thicker size bearing exchanges were promptly performed before metallosis and loosening of components. Continual examination is important to mobile bearing type of UKA because slight or less symptoms may disclose such unique dislocation. One case showed malrotation of the femoral component on 3D image. Anteroposterior view hardly disclosed the malrotation of the femoral component. Epicondylar view is an indispensable view of importance, and it can demonstrate the rotation of the femoral component. The the femoral distal end is wedge shaped and is wider posteriorly. If the femoral component is set according to the shape of medial condyle, the femoral component shifts to medial site compared with tibial component in flexion. It can account for such rare dislocation as follows. If excessive force applies on most medial side of the bearing during flexion, the lateral part of the bearing pops and the force squeezes it laterally simultaneously. Finally, the bearing jumps onto the lateral wall of the tibial component.

## 1. Introduction

Meniscal bearing type of knee prosthesis was designed to reduce contact stress between the femoral component and the bearing because the meniscal bearing automatically realigns the femorotibial relationship and functions as “check valve” for excessive rotational force between both components. On the contrary, excess mobility due to thinner bearing selection in surgery, “wear-out” of polyethylene for many years of use, or ligament deterioration may lead to dislocation of the bearing. Dislocation of the bearing promptly requires reposition because it may lead to metallosis and polyethylene wear. Dislocation of the bearing in total knee arthroplasty (TKA) is well documented [1, 2].

Unicompartmental knee arthroplasty (UKA) has remained popular with many surgeons as the less invasive surgery. For the spherical femoral component and the highly congruous meniscal bearing, Oxford Partial Knee (Biomet Inc., Swindon, UK) has a wide permissive range of implant placement and low potential of polyethylene wear [3] but has a risk of dislocation of meniscal bearing compared with fixed type. This presents warning cases that lateral dislocation of meniscal bearing without remarkable symptom was detected at periodic radiographic examination.

## 2. Report of the Cases

### 2.1. Case 1 (76-Year-Old Female)

**Figure 1:**
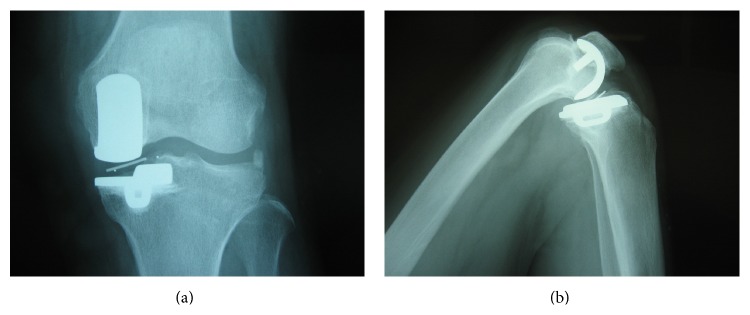
Anteropoasterior (a) weigh-bearing radiograph and lateral (b) radiograph in knee flexion showing the abnormal position of marker in meniscal bearing (case 1).

**Figure 2:**
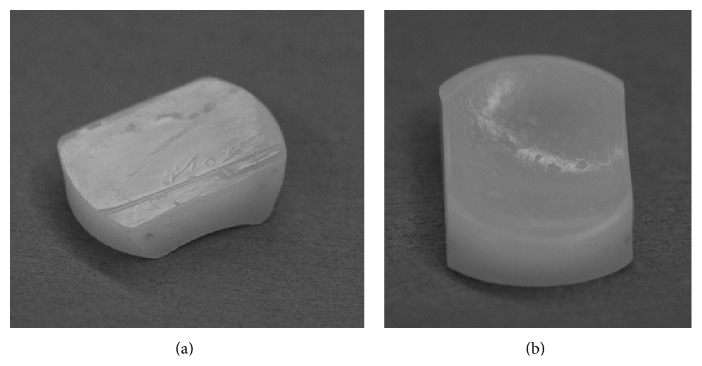
Retrieved bearing of case 1. There is a clear gutter implying that dislocation had run onto the wall for a while in the distal surface (a). There is multiple pockmarked wear in the proximal surface (b).

Seventy-year-old female suffered from medial type of primary osteoarthritis on her left knee. She underwent UKA (Biomet Oxford Partial Knee; femur XS, tibia AA, bearing 4) in September 2004 by the first author (TF). All implant sizes and positions were satisfying based on the booklet of Biomet (Oxford Microplasty Surgical Technique) except for slight overflexion (5.57°) of femoral component. Soft tissue balancing was proved to be good by stress radiography and epicondylar view. She was satisfied with her postoperative condition. At periodic (fifth year postoperatively) examination, her condition was good with normal gait and free from pain (HSS scale: 79 pts, ROM: Flex./Ext. 130°/0°). However, radiograph showed that the meniscal bearing was dislocated laterally and jumped onto the wall of tibial component (Figures 1(a) and 1(b)). At first she had no episode of the dislocation. We questioned closely and she remembered sudden pain during deep flexion for standing up in Japanese room (Tatami room) 6 months ago, four times of click and slight locking (easily unlocked) since then. We promptly performed the replacement of the meniscal bearing. There was neither abnormal synovitis nor loosening of components. The bearing was easily repositioned and dislocated. The bearing was able to be removed without hurdle. The medial collateral ligament looked intact. However, the gap seemed to be wider for size 4 bearing and thicker bearing (size 6) was chosen. Retrieved meniscal bearing demonstrated multiple pockmarked wear in both surfaces and gutter against wall of tibial component in the distal surface (Figures 2(a) and 2(b)). Postoperative condition was satisfying without pain and gait disturbance. HSS scale was improved from 79 to 92 points. ROM was improved from 130°/0° to 136°/0°. Periodic medical examination has continued and no serious problem such as loosening or dislocation was disclosed by 2014.

### 2.2. Case 2 (61-Year-Old Male)

**Figure 3:**
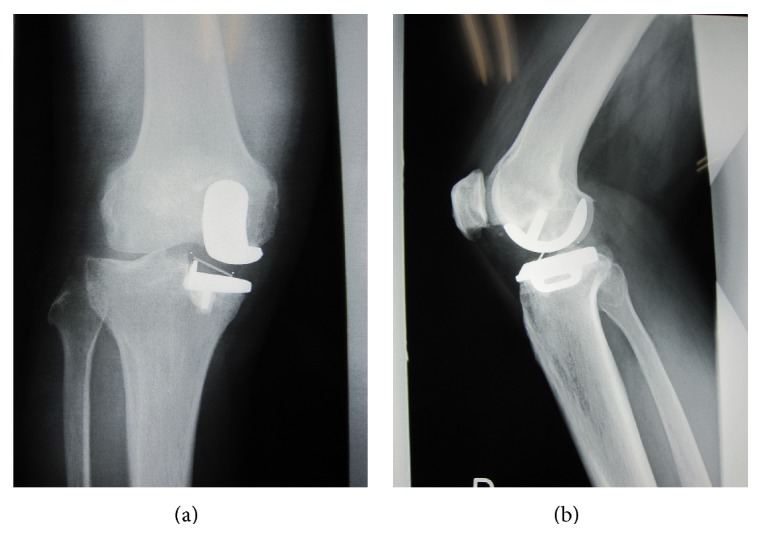
Anteroposterior (a) and lateral (b) weight-bearing radiographs showing the abnormal position of marker in meniscal bearing (case 2).

**Figure 4:**
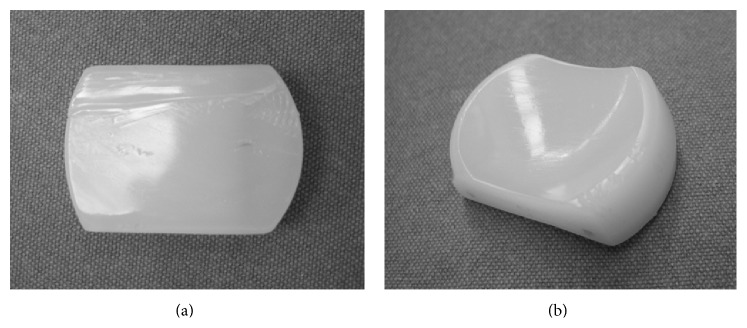
Retrieved bearing of case 2. There is a clear gutter implying dislocation in the distal surface (a). There is jagged damage of anterior lateral corner of proximal surface (b).

He underwent UKA (Biomet Oxford Partial Knee; femur S, tibia A, bearing 5) in 2005 by the 5th author (Yoshinori Kadoya) due to spontaneous osteonecrosis of his right knee. All implant sizes and positions were good based on the booklet of Biomet (Oxford Microplasty Surgical Technique). Varus-valgus instability was not detected in manual examination. He was satisfied with his postoperative condition as well as case 1. He fell into the ground and hit his right knee in 2009. He could walk normally but complained unusual feeling during motion. He came to the hospital 2 weeks later. The examination demonstrated the poor arch (ROM: Flex./Ext. 90°/−10°). Knee society score was 64 pts. Radiograph showed that the meniscal bearing was dislocated laterally and jumped onto the wall of tibial component, completely the same as case 1 (Figures 3(a) and 3(b)). Surgical assessment was performed immediately. The bearing was repositioned and we confirmed it was not dislocated easily. There was no abnormal synovitis and the medial collateral ligament looked intact. Thicker bearing (size 7) was chosen for more secure stability. Retrieved meniscal bearing demonstrated gutter against wall of tibial component as well as case 1 and additionally jagged damage of anterior lateral corner of proximal surface (Figures 4(a) and 4(b)). Postoperative condition was satisfying without pain and gait disturbance. Knee society score was improved from 64 to 100 pts. ROM was improved from 90°/−10° to 140°/0° in 2014 in spite of slight anterior knee pain.

The patients were informed that data from these cases would be submitted for publication and gave their consents, respectively.

## 3. Discussion

Lateral dislocation of the meniscal bearing could be disclosed at long term postoperative examination (case 1: 5 years, case 2: 4 years) after mobile type UKA, and the thicker bearing was replaced before the metallosis or loosening happens in both cases.

There were several reports of meniscal bearing dislocation of UKA in the past literature. All implants were Oxford Partial Knees (mobile type bearing). Dislocation rate is 0.9% to 4.0% [4–8], and this meant that mobile UKA closely related to bearing dislocation. Anterior dislocation [4, 5] was treated by closed reposition or bearing replacement. Posterior dislocation [5] was treated by surgical intervention (bearing replacement).

Symptom of anterior and posterior bearing dislocation might be more severe than lateral dislocation, because the bearing under the anterior and posterior dislocation completely falls from joint space; the bearing under lateral dislocation remains at the joint space. Patients with lateral dislocation could walk and had had their daily lives unaware of dislocation. Retrieved bearing showed the clear gutter on surface implying that the dislocated bearing had jumped onto the wall and been used in daily living for a while.Periodic radiographic examination is indispensable to disclose the lateral dislocation of the bearing soon after the incidence, because the delayed diagnosis may easily lead to metallosis and loosening of components.

**Figure 5:**
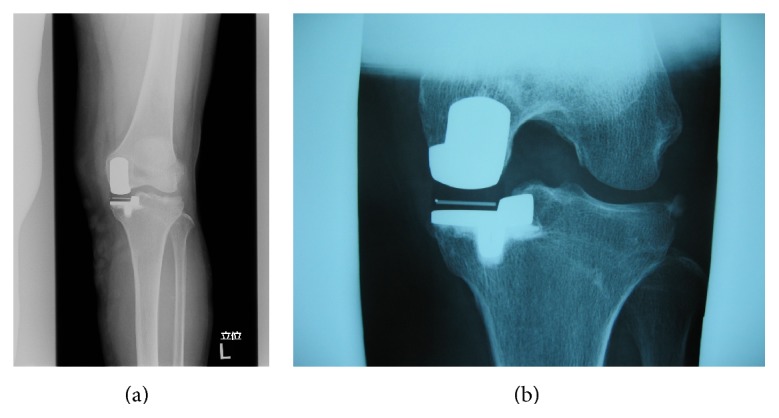
Malrotation of femoral rotation (simulation on Athena). The simulated malrotation seemed to be satisfying on simulated anterolateral view (a), although the femoral component (contour) is set according to the shape of the medial condyle on axial view of CT scan (b). (This sample is neither of the cases.)

**Figure 6:**
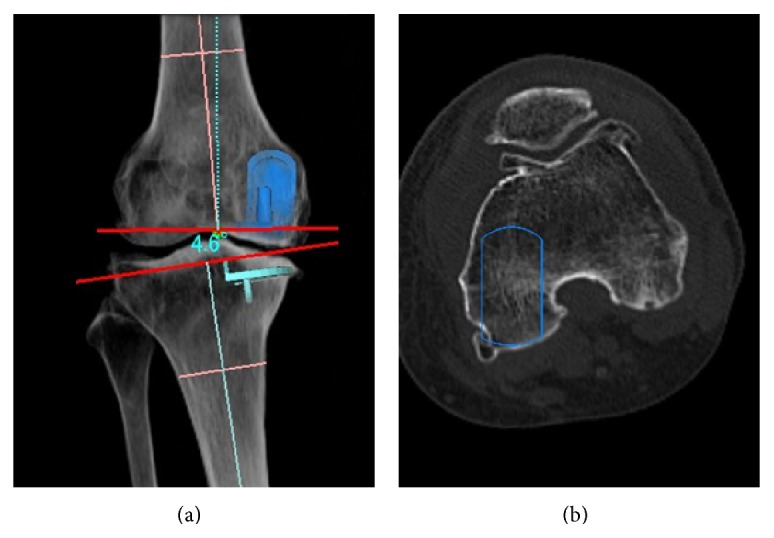
AP and epicondylar view of case 1. Although the axis of the femoral component was parallel to the tibia (a), the femoral component was set according to the axis of the medial condyle and demonstrated malrotation (b).

Preoperative soft tissue condition including ligaments was eligible for mobile bearing Oxford UKA; however, the chronic ligamentous laxity potentially existed in both cases, which related to this situation. Only laxity cannot account for the mechanism of lateral bearing dislocation. In addition, anteroposterior view hardly disclosed the malrotation of the femoral component.  Figure 5 demonstrated the difficulty to detect rotational malalignment of the femoral component in anteroposterior view on 3D digital template system (Athena, SoftCube Co., Ltd., Osaka, Japan). Epicondylar view is an indispensable view of importance for mobile type UKA, and it demonstrated the malrotation of the femoral component in case 1 (Figure 6). The femoral distal end is wedge shaped (“inverted v” shape) and is wider posteriorly. The medial meniscus and MCL prevent the medial condyle from shifting medially in normal anatomy. If the femoral component is set according to the shape of medial condyle, the medial condyle shifts to medial site compared with tibial component in flexion. The mechanism of lateral dislocation is hypothesized as follows. If excessive force applies on most medial side of polyethylene bearing during flexion, the lateral part of the bearing pops and the force squeezes the bearing laterally simultaneously. Finally, the bearing jumps onto the lateral wall of the tibia component.

There is less information about lateral dislocation of the meniscal bearing of UKA in the literature. Closed reposition may be optional. However dislocation without robust force means easy redislocation as described in case 1. We finally selected the surgical treatment and exchanged thicker bearings in both cases considering the possibility of bearing damage, and the tibial tray could be preserved.

In summary, we encountered atypical lateral dislocations of meniscal bearing in UKA without manifest symptoms. We could find the dislocation due to the continual radiographic examination, and the bearing exchange was performed before metallosis and loosening of components. Continual examination is important to meniscal bearing type of UKA for minimizing invasive revision surgery. Epicondylar view is easy to assess the rotational alignment.
